# Ultrasound Microbubbles Enhance the Efficacy of Insulin-Like Growth Factor-1 Therapy for the Treatment of Noise-Induced Hearing Loss

**DOI:** 10.3390/molecules26123626

**Published:** 2021-06-13

**Authors:** Yi-Chun Lin, Yuan-Yung Lin, Hsin-Chien Chen, Chao-Yin Kuo, Ai-Ho Liao, Ying-Liang Chou, Chia-Lien Hung, Cheng-Ping Shih, Chih-Hung Wang

**Affiliations:** 1 Graduate Institute of Medical Sciences, National Defense Medical Center, Taipei 11490, Taiwan; lyc_1023@yahoo.com.tw (Y.-C.L.); yking1109@gmail.com (Y.-Y.L.); 2 Department of Otolaryngology-Head and Neck Surgery, Tri-Service General Hospital, National Defense Medical Center, Taipei 11490, Taiwan; acolufreia@yahoo.com.tw (H.-C.C.); chefsketchup@hotmail.com (C.-Y.K.); entchou@gmail.com (Y.-L.C.); 3 Graduate Institute of Biomedical Engineering, National Taiwan University of Science and Technology, Taipei 10607, Taiwan; aiho@mail.ntust.edu.tw; 4 Department of Biomedical Engineering, National Defense Medical Center, Taipei 11490, Taiwan; 5 Department of Otorhinolaryngology, Taichung Armed Forces General Hospital, Taichung 41168, Taiwan; 6 Department of Medical Education and Research, Taichung Armed Forces General Hospital, Taichung 41168, Taiwan; chia-lien@803.org.tw; 7 Graduate Institute of Microbiology and Immunology, National Defense Medical Center, Taipei 11490, Taiwan

**Keywords:** microbubble, ultrasound, inner ear, drug delivery, insulin-like growth factor-1, noise-induced hearing loss

## Abstract

The application of insulin-like growth factor 1 (IGF-1) to the round window membrane (RWM) is an emerging treatment for inner ear diseases. RWM permeability is the key factor for efficient IGF-1 delivery. Ultrasound microbubbles (USMBs) can increase drug permeation through the RWM. In the present study, the enhancing effect of USMBs on the efficacy of IGF-1 application and the treatment effect of USMB-mediated IGF-1 delivery for noise-induced hearing loss (NIHL) were investigated. Forty-seven guinea pigs were assigned to three groups: the USM group, which received local application of recombinant human IGF-1 (rhIGF-1, 10 µg/µL) following application of USMBs to the RWM; the RWS group, which received IGF-1 application alone; and the saline-treated group. The perilymphatic concentration of rhIGF-1 in the USM group was 1.95- and 1.67- fold of that in the RWS group, 2 and 24 h after treatment, respectively. After 5 h of 118 dB SPL noise exposure, the USM group had the lowest threshold shift in auditory brainstem response, least loss of cochlear outer hair cells, and least reduction in the number of synaptic ribbons on postexposure day 28 among the three groups. The combination of USMB and IGF-1 led to a better therapeutic response to NIHL. Two hours after treatment, the USM group had significantly higher levels of Akt1 and Mapk3 gene expression than the other two groups. The most intense immunostaining for phosphor-AKT and phospho-ERK1/2 was detected in the cochlea in the USM group. These results suggested that USMB can be applied to enhance the efficacy of IGF-1 therapy in the treatment of inner ear diseases.

## 1. Introduction

The inner ear is a vulnerable sensory organ responsible for hearing and balance. In adult mammals, since cochlear hair cells have no capacity for regeneration, damage to the cochlea often results in permanent hearing loss [1]. Therefore, immediate and efficient therapy plays a critical role in achieving better hearing recovery in the context of inner ear disease. Intratympanic delivery of drugs is commonly utilized to treat several hearing and vestibular diseases [2]. Drug delivery via this approach is mainly dependent on drug diffusion through the round window membrane (RWM), an interface between the middle ear and inner ear. Therefore, the RWM is regarded as a major epithelial barrier to drug delivery [2]. RWM permeability is related to the size and electrical charge of the applied drugs [3,4]. Compared to high molecular weight substances, low molecular weight substances (molecular weight < 1000 Daltons) are more quickly transported into the inner ear through the RWM [4]. Due to the limited and selective permeability of the RWM, several delivery systems and drug carriers have been designed to improve intratympanic delivery by increasing the contact time at the RWM or enhancing drug transport through the RWM [5,6]. Ultrasound microbubbles (USMBs) were originally utilized for diagnostic imaging, and the further usefulness of this technique for inner ear drug delivery has been demonstrated [7,8,9]. The inertial and stable cavitation effects of ultrasound irradiation of microbubbles (MBs) can result in microstreaming and shock waves in the middle ear to facilitate the entry of drugs into the inner ear through the RWM. USMBs promote the local delivery of dexamethasone, a commonly administered drug, to the inner ear and then enhance its anti-inflammatory effect on the cochlea [10]. This suggests that USMBs can be used in combination with intratympanic steroid injections as a treatment modality to improve the outcomes of inner ear diseases. The changes in RWM permeability are dependent on the course of USMB exposure. Our previous results reveal that enhanced permeability of the RWM to biotin-FITC can be sustained for approximately 72 h after USMB exposure [8]. USMB is a safe, less invasive system for inner ear drug delivery, and no complications of hearing loss or cochlear damage have been reported [7,9].

Insulin-like growth factor 1 (IGF-1) is a polypeptide of 70 amino acids with a molecular weight of 7649 Daltons and an important modulator of neurogenesis and hearing development [11,12]. It has been recognized as a novel therapeutic agent for inner ear diseases [13]. In a cochlear explant culture model of aminoglycoside-induced ototoxicity, IGF-1 treatment protects cochlear hair cells from apoptosis and induces the proliferation of supporting cells [14,15]. IGF-1 also induces the regeneration of degenerated synapses between inner hair cells and spiral ganglion neurons [16]. In animal studies, the intratympanic delivery of IGF-1 has preventive and therapeutic effects on noise-induced hearing loss (NIHL) and ischemic cochlear damage [17,18,19]. Clinical studies have revealed that local IGF-1 administration to the round window niche via gelatin hydrogel can improve hearing levels in patients with sudden sensorineural hearing loss refractory to systemic steroids [20,21,22]. In terms of the size of delivered substances, the RWM is more permeable to smaller substances [4]. IGF-1 has a larger molecular weight than low molecular weight drugs, such as dexamethasone and antibiotics, which may restrain its diffusion through the RWM. We hypothesized that USMBs can enhance the ability of IGF-1 to pass through the RWM and thus improve the therapeutic effect of IGF-1. To verify this hypothesis, the present study aimed to investigate the enhancing effect of USMBs on IGF-1 delivery to the inner ear. Moreover, USMB-mediated IGF-1 treatment was administered after noise exposure. The improvement in hearing, alleviation of structural damage and changes in IGF-1 signaling were further explored in an animal model of NIHL.

## 2. Results

### 2.1. USMBs Enhanced the Delivery Efficiency of IGF-1 to the Inner Ear

**Figure 1:**
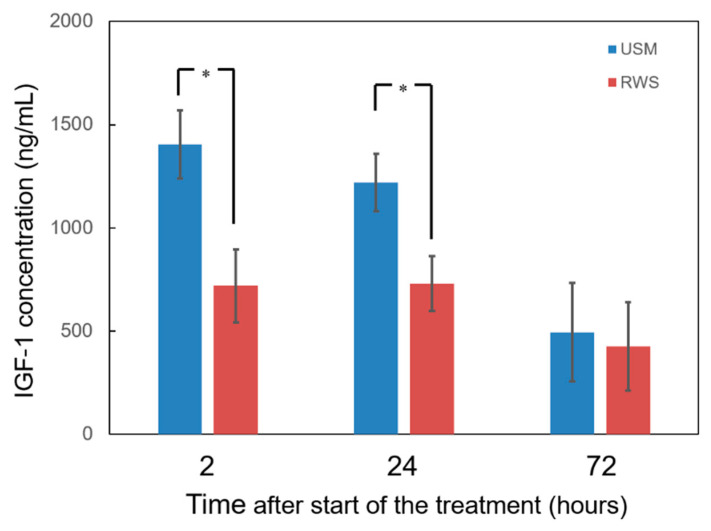
Concentration of IGF-1 in the perilymphatic fluid of the inner ear following IGF-1 treatment with and without ultrasound microbubbles (USMBs). In the USM group, IGF-1 was delivered following USMB treatment. The results are expressed as the mean ± SEM; n = 6 for each bar; * *p* < 0.05. IGF-1, insulin-like growth factor-1; USM, ultrasound microbubble treatment; RWS, round window soaking.

To determine whether the application of ultrasound-irradiated MBs facilitates the delivery of IGF-1 to the inner ear, the IGF-1 concentration in the inner ear perilymph in the USM group, which received local application of rhIGF-1 to the RWM following USMB treatment, and the RWS group, which received local application of rhIGF-1 without USMBs, were compared at different posttreatment time points. As shown in Figure 1, the inner ear concentration of rhIGF-1 in the USM group was significantly higher than that in the RWS group 2 and 24 h after treatment (2 h after treatment: 1405 ± 165.4 ng/mL vs. 719 ± 175.3 ng/mL, *p* = 0.017; 24 h after treatment: 1219 ± 138.9 ng/mL vs. 731 ± 133.6 ng/mL, *p* = 0.03). However, there was no significant difference in rhIGF-1 levels between the two groups 72 h after treatment. These findings demonstrated that USMBs can facilitate IGF- 1 delivery to the inner ear for at least 24 h by enhancing RWM permeability. Following USMB treatment, the IGF-1 level in the inner ear was increased by 1.95- and 1.67-fold 2 and 24 h posttreatment, respectively.

### 2.2. The Combination of USMBs and IGF-1 Had a Better Therapeutic Effect on NIHL

**Figure 2:**
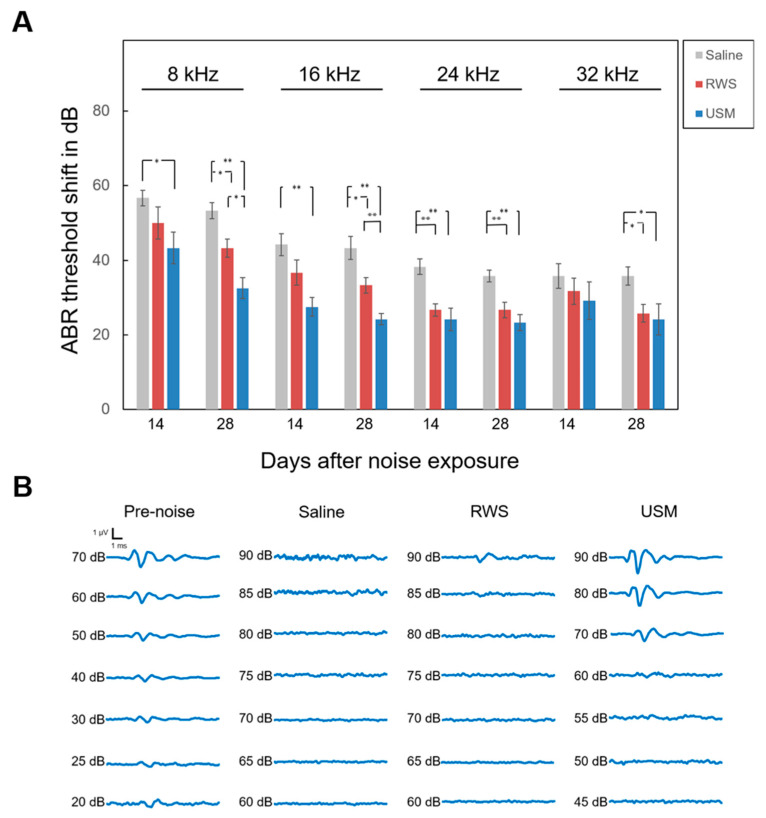
Evaluation of the therapeutic effects of IGF-1 on hearing loss after noise exposure by ABR assessment. (**A**) The ABR threshold shift after noise exposure was compared between the saline-treated, RWS (IGF-1 treatment without USMBs), and USM (IGF-1 treatment with USMBs) groups. The results are expressed as the mean ± SEM; n = 6 for each bar; * *p* < 0.05, ** *p* < 0.01. (**B**) Representative ABR response waveforms to 8 kHz stimuli for the three groups before and 28 days after noise exposure. ABR, auditory brainstem response; IGF-1, insulin-like growth factor-1; USM, ultrasound microbubble treatment; RWS, round window soaking.

**Figure 3:**
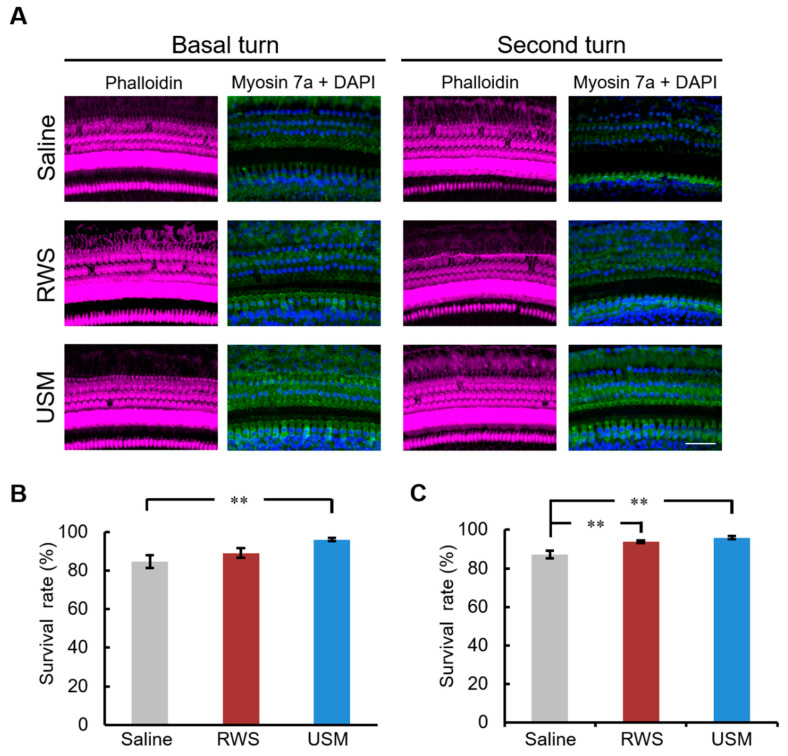
IGF-1 delivery aided by ultrasound microbubbles enhances the preservation of cochlear hair cells following noise exposure. (**A**) Representative images of a surface preparation of the basal and second turns of the cochlea from the three groups on day 28 after noise exposure. Immunofluorescence staining showing cell bodies of hair cells (green, myosin 7a), nuclei (blue, DAPI) and filamentous actin (magenta, phalloidin). This experiments were repeated six times. Scale bars = 50 μm. The survival rates of the outer hair cells in the basal turn (**B**) and second turn (**C**) in guinea pigs from each experimental group. The results are expressed as the mean ± SEM; n = 6 for each bar; ** *p* < 0.01. IGF-1, insulin-like growth factor-1; DAPI = 4,6-diamidino-2-phenylindole; USM, ultrasound microbubble; RWS, round window soaking.

**Figure 4:**
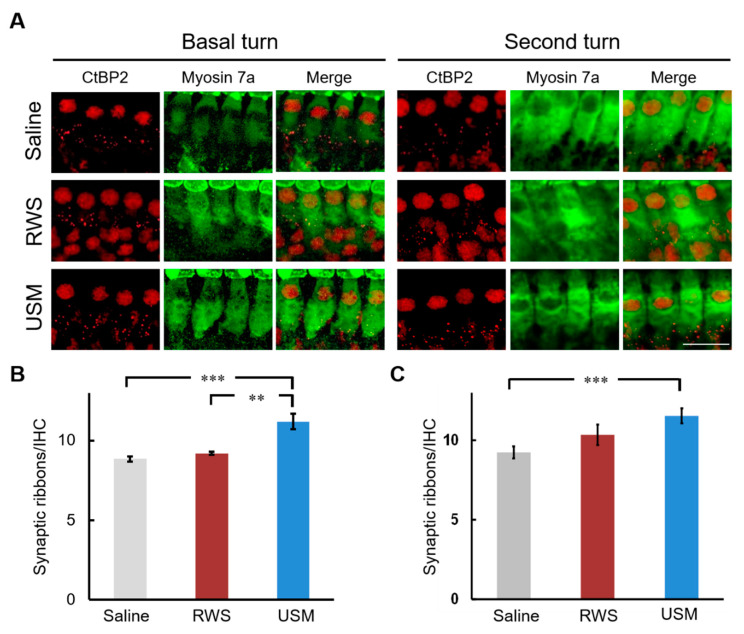
IGF-1 delivery aided by ultrasound microbubbles attenuates the reduction in the number of synaptic ribbons following noise exposure. (**A**) Representative images of inner hair cells in the basal and second turns of the cochlea in the three groups on day 28 after noise exposure. Immunofluorescence staining showing synaptic ribbons (red, ctbp2) and cell bodies of hair cells (green, myosin 7a). This experiment was repeated six times. Scale bars = 100 μm. The number of synaptic ribbons in inner hair cells of the basal turn (**B**) and second turn (**C**) was compared between each experimental group. The results are expressed as the mean ± SEM; n = 6 for each bar; ** *p* < 0.01, *** *p* < 0.001. IGF-1, insulin-like growth factor-1; USM, ultrasound microbubble; RWS, round window soaking.

To investigate whether USMBs can strengthen the therapeutic effect of IGF-1 on NIHL, USMBs followed by rhIGF-1 were administered to the USM group, while IGF-1 only was administered to the RWS group 24 h after noise exposure. ABRs were recorded prior to and 14 and 28 days after noise exposure (Table 1 and Figure 2). On days 14 and 28 after noise exposure, considerable differences in the ABR threshold shift were found between the USM and saline groups following exposure to the 8-, 16- and 24-kHz stimuli. A significant difference in threshold shift at 32 kHz was found between the USM and saline groups on day 28 after noise exposure. The USM group had a lower threshold shift than the saline group following noise exposure. Compared to the RWS group, the USM group had significantly lower threshold shifts at 8 kHz and 16 kHz on day 28 after noise exposure. Overall, the USM group exhibited the best hearing recovery among the three groups following noise exposure. Subsequently, the loss of outer hair cells and the number of synaptic ribbons in the cochlea following noise exposure and treatment were evaluated. Figure 3 shows the severity of outer hair cell loss in the cochleae of the three groups on day 28 after noise exposure. Myosin 7a immunostaining was used to identify hair cells. The survival rates of the outer hair cells in the three groups were as follows: 84.8% ± 3.31% in the basal turn of saline group, 87.1% ± 1.97% in the second turn of saline group, 89.1% ± 2.49% in the basal turn of RWS group, 93.9% ± 0.7% in the second turn of RWS group, 96.1% ± 0.82% in the basal turn of USM group, and 96% ± 0.87% in the second turn of USM group. In comparison of the survival rates of the three groups in the basal turn, the USM group had a higher survival rate than the saline group (*p* = 0.007). The survival rate of the RWS group was not significantly different from that of the saline and USM groups (RWS vs. saline, *p* = 0.448; RWS vs. USM, *p* = 0.109). In comparison of the survival rates of the three groups in the second turn, the USM and RWS groups had a higher survival rate than the saline group (USM vs. saline, *p* = 0.002; RWS vs. saline, *p* = 0.006). There was no significant difference in the survival rate between the USM and RWS groups (*p* = 0.617). Overall, there was a prominent loss of outer hair cells in the organ of Corti in the saline group. Compared to the saline group, the USM group had more preservation of outer hair cells in the basal and second turns, but the RWS group only preserved more in the basal turn. These results suggested that USMBs followed by IGF-1 treatment led to good preservation of outer hair cells from noise damage. Figure 4 shows the presence of synaptic ribbons in inner hair cells in the three groups on day 28 after-noise exposure. The number of synaptic ribbons in the three groups were as follows: 8.7 ± 0.17 in the basal turn of saline group, 9.2 ± 0.37 in the second turn of saline group, 9.2 ± 0.09 in the basal turn of RWS group, 10.3 ± 0.64 in the second turn of RWS group, 11.2 ± 0.5 in the basal turn of USM group and 11.5 ± 0.48 in the second turn of USM group. The saline group had the fewest synaptic ribbons in inner hair cells among the three groups. In the basal turn, the USM group, which had the largest number of synaptic ribbons, exhibited more significant restoration of synaptic ribbons than the RWS and saline groups (USM vs. saline, *p* < 0.001; USM vs. RWS, *p* = 0.002) (Figure 4B). The number of synaptic ribbons of the RWS group in the basal turn was not significantly different from that of the saline group (*p* = 0.133). In the second turn, there was a significant difference in the number of synaptic ribbons, as the USM group had more synaptic ribbons than the saline group (*p* < 0.001) (Figure 4C). The number of synaptic ribbons of the RWS group in the second turn was not significantly different from that of the saline groups (*p* = 0.325). These findings suggested that USMBs improved the efficacy of topical administration of IGF-1 for the treatment of NIHL and that this combination resulted in a superior therapeutic effect against noise-induced cochlear damage, including the loss of outer hair cells and a reduction in the number of synaptic ribbons.

### 2.3. USMBs Enhanced the Therapeutic Effect of IGF-1 by Promoting Activation of IGF-1 Signaling Pathways in the Cochlea

**Figure 5:**
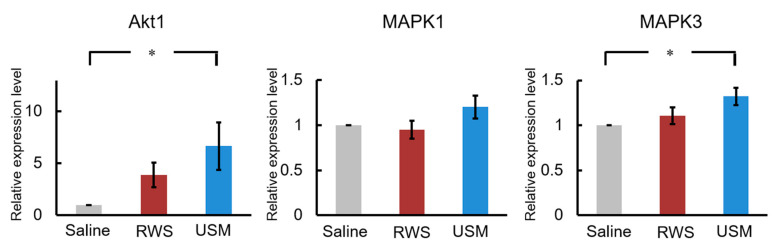
Ultrasound microbubble-mediated IGF-1 treatment increases the mRNA expression of Akt1 and Mapk3 in the cochlea after noise exposure. The combination of ultrasound microbubbles and IGF-1 (USM group), IGF-1 only (RWS group) or saline only (saline group) was administered 24 h after noise exposure. Total RNA was isolated from whole cochleae from the three groups 2 h after treatment. Akt1, Mapk1, and Mapk3 expression levels were quantified by quantitative PCR. The results are expressed as the mean ± SEM; n = 9 for each bar; * *p* < 0.05. IGF-1, insulin-like growth factor-1; USM, ultrasound microbubble; RWS, round window soaking.

**Figure 6:**
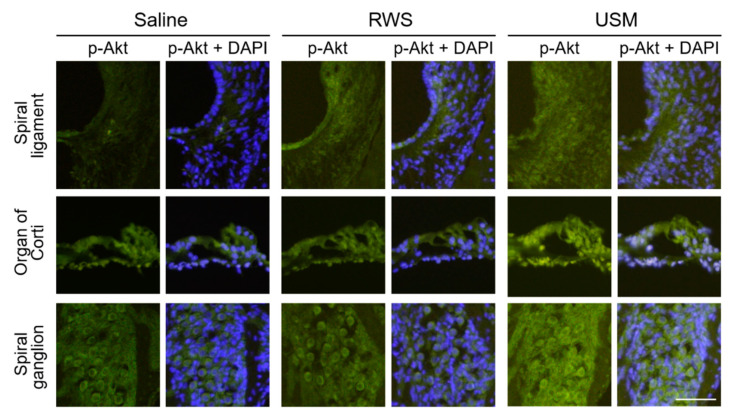
Immunohistochemical staining for p-AKT in a cryosection of the cochlea after noise exposure and different treatments. The combination of ultrasound microbubbles and IGF-1 (USM group), IGF-1 only (RWS group) or saline only (saline group) was administered 24 h after noise exposure. The samples were collected 2 h after treatment. Immunofluorescence staining showing p-AKT immunostaining (green) and nuclei (blue, DAPI). This experiment was repeated four times. Scale bars = 100 μm. IGF-1, insulin-like growth factor-1; DAPI = 4,6-diamidino-2-phenylindole; USM, ultrasound microbubble; RWS, round window soaking.

**Figure 7:**
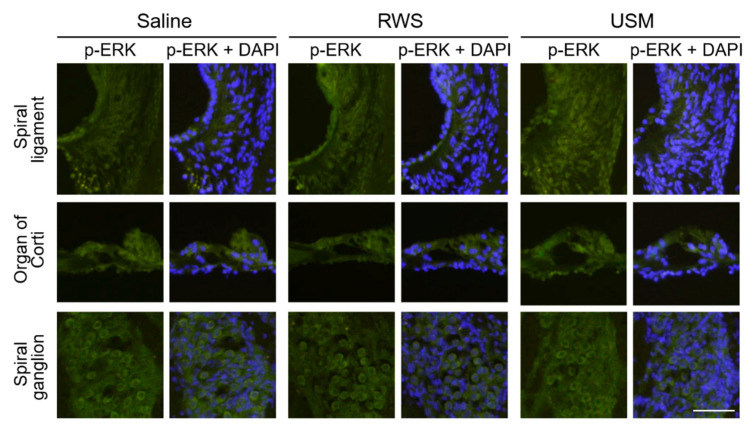
Immunohistochemical staining for p-ERK 1/2 in a cryosection of the cochlea after noise exposure and different treatments. The combination of ultrasound microbubbles and IGF-1 (USM group), IGF-1 only (RWS group) or saline only (saline group) was administered 24 h after noise exposure. The samples were collected 2 h after treatment. Immunofluorescence staining showing p-ERK immunostaining (green) and nuclei (blue, DAPI). This experiment was repeated four times. Scale bars = 100 μm. IGF-1, insulin-like growth factor-1; DAPI = 4,6-diamidino-2-phenylindole; USM, ultrasound microbubble; RWS, round window soaking.

To further explore the cochlear IGF-1 signaling in the NIHL model after USMB-mediated rhIGF-1 treatment, the gene expression and activation of protein kinases related to noise-induced damage of the cochlea were compared between the three groups 2 h after treatment. Figure 5 shows the relative gene expression levels of Akt1, Mapk1 and Mapk3 in the three groups. The levels of Akt1 and Mapk3 mRNA in the USM group were 6.7- and 1.3-fold those in the saline group, respectively. The USM group had significantly higher gene expression levels of Akt1 and Mapk3 than the saline group (Akt1: *p* = 0.041; Mapk3: *p* = 0.025). Compared to the saline group, the RWS group exhibited no significant difference in the mRNA levels of Akt1 and Mapk3. There was no significant difference in the mRNA level of Mapk1 between the USM, RWS, and saline groups. The phosphorylation levels of AKT and ERK 1/2 in the cochleae of the three groups were evaluated 2 h after treatment and are shown in Figure 6 and Figure 7. The USM group had more intense immunostaining for p-AKT in the spiral ligament, organ of Corti, and spiral ganglion than the RWS and saline groups (Figure 6). The RWS group had stronger p-AKT staining in the spiral ligament than the saline group. The difference in the intensity of p-ERK 1/2 staining between the three groups was assessed in the cochlea (Figure 7). The USM group had the most intense p-ERK staining in the spiral ligament, followed by the RWS and saline groups. These results indicated that USMB enhanced the efficacy of IGF-1 treatment by improving IGF-1 delivery and promoting signaling pathways downstream of IGF-1 in the cochlea.

## 3. Discussion

In the present study, the rhIGF-1 level in the inner ear was increased by 1.95- and 1.67-fold 2 and 24 h after USMB and local rhIGF-1 application, respectively. The enhancing effect of USMBs on rhIGF-1 delivery to the inner ear persisted for at least 24 h. This suggests that the USMB-induced sonoporation effect promotes the passage of IGF-1 through the RWM by enhancing RWM permeability. During ultrasound insonation, the induced cavitation of MBs can exert sonoporation effects on the targeted cells and region [23]. The biophysical progress of sonoporation not only results in pore formation in the cell membrane but may also be involved in intracellular responses, including cytoskeletal disassembly, cell nucleus contraction, and intracellular transport [24,25,26]. Endocytosis and cytoskeletal rearrangement induced by sonoporation promote intracellular transport [23,27]. Sonoporation increases the permeability of the targeted region and thus facilitates the delivery of genes and drugs [23]. The outer epithelial layer of the RWM is the main barrier of substance passage from the middle ear to the inner ear [3,4,6,8]. Moreover, the outer epithelial cells have microvilli and abundant organelles to execute intracellular transport of substances [3]. Our previous study revealed ultrastructural changes in the RWM after USMB exposure and the direct effect of cavitation-enhanced sonoporation on the RWM [8]. The sonoporation-induced enhancement of RWM permeability is strongly related to the formation of heterogeneous pore-like openings and disruption of the outer epithelial layer of the RWM. Interestingly, because the continuous basement membrane of the outer epithelial layer is preserved after USMB exposure, the defect in the RWM is subsequently recovered. Therefore, the use of USMBs is a promising method for promoting the delivery of therapeutic agents to the inner ear [7,10]. The present study is the first to demonstrate that the delivery efficiency of IGF-1 into the inner ear can be increased by USMBs. The major mechanism responsible for enhancing IGF-1 delivery by USMBs is pore formation and disruptions on the outer epithelial layer which promote IGF-1 passage across the RWM. Other possible mechanisms include sonoporation-induced endocytosis and cytoskeletal rearrangement in outer epithelial cells which can enhance intracellular transport.

IGF-1 plays a key role in the development, growth and differentiation of the inner ear [28]. IGF-1 knockout mice present with severe sensorineural hearing loss, associated with abnormal cochlear morphology and degeneration of the spiral ganglion [28]. IGF-1 treatment enhances the phosphorylation of AKT and ERK and the expression of related genes. When IGF-1 binds to the IGF-1 receptor, downstream signaling pathways, including two canonical phosphatidylinositol 3-kinase/protein kinase B (PI3K/AKT) and Ras/MAPK pathways and some accessory pathways, are activated [29]. In aminoglycoside-damaged cochleae, IGF-1 treatment activates the PI3K/AKT and Ras/MAPK pathways to inhibit the apoptosis of inner hair cells and to promote the proliferation of supporting cells for the maintenance of outer hair cells [14,15]. Gap43 and Ntn1 play an important role in hair cell protection in neomycin-damaged cochleae, and their expression can be upregulated by IGF-1 [30,31]. The activation of AKT and ERK is related to susceptibility to NIHL [32,33]. PI3K/AKT signaling is inhibited after noise exposure, and Akt1 knockout mice have increased sensitivity to NIHL [32]. The administration of activated protein C induces phosphorylation of AKT and has a protective effect on NIHL [34]. Although there is some controversy about the role of p-ERK1/2 in NIHL, some studies suggest that phosphorylation of ERK1/2 results in a protective response to NIHL [33,35]. Cochlear synaptopathy, a form of damage to the synapses between inner hair cells and spiral ganglion neurons, is one of the main lesions contributing to NIHL [36]. Inhibition of the IGF-1 receptor results in a decrease in the number of synaptic ribbons of inner hair cells in cochlear explant cultures, and synaptic ribbons are spontaneously recovered without inhibition of the IGF-1 receptor [37]. Treatment with exogenous IGF-1 promotes the regeneration of synaptic ribbons. In excitatory amino acid-damaged cochlear explants, the application of IGF-1 induces an increase in the number of synapses between inner hair cells and spiral ganglion neurons [16]. This demonstrates that IGF-1 treatment can facilitate synaptic regeneration of the damaged cochlea. The present study revealed that the combination of USMB treatment and local IGF-1 delivery had a better therapeutic effect on NIHL than IGF-1 treatment alone. Treatment with USMBs and IGF-1 resulted in better hearing recovery, less loss of outer hair cells, and more preservation of synaptic ribbons after acoustic trauma. Moreover, we found that the combination of USMBs and IGF-1 induced the gene expression of Akt-1 and Mapk3 and phosphorylation of AKT and ERK 2 h after treatment. Therefore, this combined treatment is an effective therapy for NIHL and has high potential for clinical application in the future.

We demonstrated that USMBs can effectively enhance IGF-1 delivery through the RWM to result in better drug action in the inner ear. Previous studies have reported that drugs and nucleic acids can be loaded on the surface of MBs via surface modification of MBs or electrostatic interactions [38,39,40]. Under ultrasound exposure, loaded MBs can enhance drug and gene delivery to targeted cells and areas. Moreover, the coating of MB with substances to be delivered can reduce the degradation of the substances [39]. IGF-1 has a positive surface charge and can be electrostatically loaded on MBs with a negative surface charge, including phospholipid-shelled MBs and albumin-shelled MBs [41,42]. Our previous study demonstrated that the transcanal approach of USMB administration is an effective method of drug delivery for the inner ear [9]. In clinical practice, the transcanal approach is often utilized for the transtympanic injection of drugs to treat various inner ear diseases. The transcanal approach has the advantages of convenience and noninvasiveness. Accordingly, application of ultrasound-irradiated IGF-1-loaded MBs via a transcanal approach may be a more practical and effective treatment modality for inner ear diseases and needs to be further investigated.

## 4. Materials and Methods

### 4.1. Animals and Study Design

Healthy pigmented guinea pigs weighing 250–350 g with a normal Preyer’s reflex were used in this study. The experimental protocol was approved by the Institutional Animal Care and Use Committee of the National Defense Medical Center, Taipei, Taiwan (protocol code: IACUC-18-063). The animal care protocols complied with institutional guidelines and regulations. A total of 47 guinea pigs were divided into several experimental groups. Both ears of each guinea pig were used in experiments. The first part of this study was performed to assess the concentration of IGF-1 in perilymphatic fluid after manipulation. In the ultrasound microbubble (USM) group, the tympanic bulla was opened and filled with 200 µL MBs. The MBs were then exposed to ultrasound for 1 min. At the end of the exposure period, the used MBs in the tympanic bulla were replaced with new MBs, and then the irradiation procedure was repeated. Ultrasound exposure was performed three times throughout the procedure. A gelatin sponge (Johnson & Johnson, New Brunswick, NJ, USA) was cut into small pieces with a size of 1.5 mm^3^, and one piece of gelatin sponge was soaked with 10 µL recombinant human IGF-1 (rhIGF-1, Biovision, Inc., Milpitas, CA, USA) at a concentration of 10 µg/µL. Then, a piece of rhIGF-1-soaked gelatin sponge was placed on the RWM. In the round window soaking (RWS) group, a piece of gelatin sponge soaked with 10 µL of rhIGF-1 (10 µg/µL) was placed on the RWM. The second part of this study investigated the therapeutic effect of local delivery of rhIGF- 1 with and without USMBs on acoustic trauma. In the saline group (the control group), a piece of gelatin sponge soaked with 10 µL saline solution was placed on the RWM 24 h after noise exposure. The same procedure was used for the USM group as that in the first part of the experiment, 24 h after noise exposure. In the RWS group, a piece of rhIGF-1-soaked gelatin sponge was placed on the RWM 24 h after noise exposure.

### 4.2. Preparation of MBs and Ultrasound Exposure

Phospholipid-shelled SonoVue MBs were used in this study. In MBs, the core is sulfur hexafluoride and the shell consists of diarachidoylphosphatidylcholine [43]. SonoVue (Bracco, Milan, Italy) was prepared as follows: 5 mL sterile 0.9% saline was added to the vial to be mixed with SonoVue lyophilisate, and then a liquid solution was formed by vigorous vibration of the vial for 20 s [44,45]. The solution had MBs with the size ranging from 0.7 to 10 μm and a concentration of 1−5 × 10^8^ bubbles/mL [43,44,45]. SonoVue solution was administered to the USM group. An ST2000V ultrasound device with a 6-mm diameter transducer was used for irradiation. Our previous study showed that an acoustic intensity of 3 W/cm^2^ for 1 min is the optimal parameter for delivering the drug to the inner ear [7]. The parameters were set as follows: intensity of 3 W/cm^2^, duty cycle of 50%, and frequency of 1 MHz. The transducer was placed 5 mm away from but facing the RWM, and sonication was performed for the indicated amount of time.

### 4.3. Surgical Technique

The surgical procedures were described in our previous study [7]. Guinea pigs were anesthetized by intramuscular injection of ketamine (Imalgene; Merial, Lyon, France) at a concentration of 80 mg/kg and xylazine (Rompun; Bayer, Leverkusen, Germany) at a concentration of 10 mg/kg and kept warm with a heating pad. A fenestration (approximately 4 mm in diameter) was made in the tympanic bulla by drilling with diamond burrs. The round window was exposed through this fenestration for the placement of drug-soaked gelatin sponges subjected or not to sonication on the RWM. All these procedures were performed under an operating microscope (F-170; Carl Zeiss, Jena, Germany). After the appropriate procedure was performed, the surgical wound was sutured in layers.

For inner ear perilymph collection, guinea pigs were immediately euthanized using CO_2_ gas. The tympanic bulla was harvested, and the cochlea was carefully exposed without damaging the ossicles and bony structure of the inner ear. A pipette with a 10 µL microtip was gently inserted through the RWM into the scala tympani for perilymph aspiration. The collected samples were then centrifuged immediately, stored at −80 °C, and later processed for the analysis of rhIGF-1 concentration.

### 4.4. Noise Exposure

Guinea pigs were anesthetized, placed between two loudspeakers in a soundproof booth and exposed for 5 h to 118 dB SPL 8 kHz narrowband noise generated by a generator (Soundcraft Compact4, Stamford, CT, USA) and amplified by a power amplifier (YAMAHA, Hamamatsu, Japan). Noise intensity was measured with a sound level meter (Rion, Tokyo, Japan). The noise level variation was less than 1 dB within the space available to the animal. The body temperature of each animal was maintained at a physiological level with a heating pad, and the animals were closely monitored throughout the entire exposure time.

### 4.5. ELISA Analysis of rhIGF-1 Levels

A human IGF-1 ELISA kit (R&D System Inc., Minneapolis, MN, USA) was used to measure rhIGF-1 levels. The rhIGF-1 solution (6 ng/mL to 0.1 ng/mL) was serially diluted for each set of perilymph samples to produce a standard concentration curve. The test and control samples, as well as the positive and negative controls provided by the manufacturer, were added to a 96-well microplate and incubated with the diluted drug-enzyme conjugate at room temperature for 1 h. The plate was then washed 3 times with diluted wash buffer to remove any unbound sample or drug-enzyme conjugate. Substrate solution was added and incubated for 30 min. The reaction was then halted with stop solution. The plate was read with an ELISA microplate reader equipped with a 450-nm filter (Synergy H4 Hybrid Reader). The absorbance value obtained by the ELISA reader was plotted and compared to the standard concentration curve to calculate the concentration of rhIGF- 1 present in each perilymph sample. In this assay, the minimal detectable perilymph concentration of rhIGF-1 was 0.1 ng/mL.

### 4.6. Quantitative PCR Analysis of Mapk1, Mapk3 and Akt1 Gene Expression

For total RNA extraction, whole cochleae were collected into tubes with MagNA lyser green beads (Roche Molecular Systems Inc., Indianapolis, IN, USA) and lysis buffer. Then, a MagNA Lyser Instrument (Roche Molecular Systems Inc., Indianapolis, IN, USA) was used for tissue disruption and homogenization of the tissues for RNA extraction. Following this, the samples were centrifuged to collect the supernatant. The supernatant was transferred to a new tube, and then a high-purity RNA isolation kit (F. Hoffmann-La Roche Ltd., Basel, Switzerland) was used. The RNA was converted to cDNA using a QuantiNova Reverse Transcription Kit (QIAGEN GmbH, Hilden, Germany). Gene expression was measured with TaqMan gene expression assays (Thermo Fisher Scientific Inc., Waltham, MA, USA) for Mapk1 (Mapk1/Erk2 gene; probe ID: Hs01046830_m1), Mapk3 (MAPK3/Erk1 gene; probe ID: Hs00385075_m1), Akt1 (Akt1 gene; probe ID: Hs00178289_m1) and Gapdh (Gapdh gene; probe ID: Hs02758991_g1) using a QuantiNova Probe RT-PCR Kit (QIAGEN GmbH, Hilden, Germany) and a QuantStudio 5 Real-Time PCR system (Thermo Fisher Scientific Inc., Waltham, MA, USA). The qPCR data are presented as the gene expression level relative to the level in the controls after normalization to the expression of GAPDH.

### 4.7. Surface Preparation of the Organ of Corti

Guinea pigs were euthanized 4 weeks after noise exposure, and the temporal bones were quickly removed. The cochlea were intrascalarly perfused with 4% paraformaldehyde in PBS. The cochleae were fixed in 4% paraformaldehyde in PBS for 1 h at room temperature. The tissues were rinsed with PBS, and the bone surrounding the organ of Corti was removed. The organ of Corti was then carefully dissected and immersed in the same fixative overnight. The tissues were then incubated with anti-carboxyl-terminal binding protein 2 (CtBP2) IgG1 (1:100; 612044, BD Biosciences, San Jose, CA, USA) and anti-myosin 7a (1:100; NB120-3481, Novus Biologicals, Littleton, CO, USA) polyclonal antibodies for 2 h. After three washes with PBS, the tissues were incubated with Alexa Fluor 555-conjugated goat anti-mouse IgG1 (γ1) (1:200; A21127, ThermoFisher Scientific, Waltham, MA, USA) and Alexa Fluor 555-conjugated goat anti-rabbit (1:500; A21428, Thermo Fisher Scientific, Eugene, OR, USA) antibodies for 1 h. The samples were incubated with 2% Alexa Fluor 647-conjugated phalloidin (A22287, Thermo Fisher Scientific, Waltham, MA, USA) for 30 min at room temperature, rinsed with PBS, mounted with DAPI Fluoromount-G^®^ mounting medium (SouthernBiotech, Birmingham, AL, USA), and covered with a coverslip for analysis. Fluorescence images were obtained using a confocal laser scanning microscope (Zeiss LSM 880, Carl Zeiss, Jena, Germany).

### 4.8. Analysis of Outer Hair Cell Survival and Synaptic Ribbon Counts

The flat prepared surface of the organ of Corti was examined with a confocal laser scanning microscope. The outer hair cell survival rate was calculated using the following formula: survival rate% = 100 × ((the number of outer hair cells present in the examined specimens)/(the number of examined specimens)/3) [27]. The z-stack images were analyzed to count synaptic ribbons. CtBP2-positive puncta in inner hair cells were identified as synaptic ribbons. The number of puncta in each inner hair cell was compared between the three groups.

### 4.9. Cochlear Cryosections and Immunostaining for Phospho-ERK1/2 and Phospho-AKT

The temporal bones were harvested and gently perfused with 4% paraformaldehyde by cochleostomies at the round window. Then, they were fixed in 4% paraformaldehyde in PBS for 2 h at room temperature and decalcified in 10% EDTA (pH = 7.3) at 4 °C for 2 weeks. The solution was changed every 2–3 days. Decalcified cochleae were immersed in 10% sucrose for 1 h, 20% sucrose for 1 h, and 30% sucrose overnight. The cochleae were sectioned at a thickness of 14 µm with a cryostat microtome after being immersed in OCT compound at room temperature overnight. Then, the samples were permeabilized with 0.3% Triton X-100 for 10 min in BlockPRO blocking buffer (Visual Protein Biotechnology, Taipei, Taiwan). After washing with PBST, the slides were incubated with 3% hydrogen peroxidase in methanol for 10 min to block endogenous peroxidase activity and then washed with PBST. Nonspecific antibody binding was blocked with BlockPRO blocking buffer for 1 h at room temperature. The slides were incubated with polyclonal primary antibodies against phospho-ERK1/2 (p-ERK1/2, 1:100; 44-680G, Thermo Fisher Scientific Inc., Waltham, MA, USA) and phospho-AKT (Ser473) (p-AKT, 1:50; 9271, Cell Signaling Technology Inc., Danvers, MA, USA) in antibody dilution buffer (Dako, Agilent Technologies Inc., Santa Clara, CA, USA) and incubated in a humidified chamber for 1 h at room temperature. After being washed with PBST, the slides were stained with an Alexa Fluor 488-conjugated donkey anti-rabbit antibody (1:500; A21206, Thermo Fisher Scientific Inc., Waltham, MA, USA) for an additional 60 min. After being washed 3 times with PBST, the slides were mounted with DAPI Fluoromount-G (Southern Biotech, Birmingham, AL, USA). The slides were examined using an Olympus BX50 microscope (Olympus Life Science, Waltham, MA, USA).

### 4.10. Auditory Brainstem Response Recording

Auditory function was assessed by recording auditory brainstem responses (ABRs) as described previously [7]. Guinea pigs were anesthetized and kept warm with a heating pad in a sound-attenuating chamber for the duration of the recording. Subdermal needle electrodes were inserted at the vertex (positive), below the pinna of the ear (negative), and on the back (ground) of each guinea pig. Specific stimuli (clicks and 8-, 16-, 32-kHz tone bursts) were produced by using SigGen software (Tucker-Davis Technologies, Gainsville, FL, USA) and delivered to the external auditory canal. The average response to 1024 stimuli at intensities ranging from 5 dB to 90 dB for each frequency was determined by reducing the sound intensity in 5-dB steps until the threshold was reached. The resulting ABR threshold was defined as the lowest intensity at which a reproducible deflection in the evoked response trace could be recognized. The threshold shift was defined as the difference in the threshold between the indicated day after noise exposure and before noise exposure.

### 4.11. Statistical Analysis

The obtained data were analyzed statistically using two-tailed Student’s t-test for comparisons between two groups. Multiple groups were compared using one-way ANOVA followed by Scheffe’s multiple-comparisons test. A probability value of <0.05 was considered indicative of a significant difference. The data are expressed as the mean ± standard error of the mean.

## 5. Conclusions

After application of USMBs to the RWM, IGF-1 was more efficiently delivered to the inner ear through the RWM. In our model of NIHL, the combination of USMBs and IGF- 1 was demonstrated to have a better treatment effect than IGF-1 alone. This suggests that USMB-mediated IGF-1 treatment is an effective therapy for inner ear disease and has high potential for clinical application in the future.

## Figures and Tables

**Table 1:** Threshold shift of ABR in the saline, RWS (round window soaking) and USM (ultrasound microbubble) groups after noise exposure.

Stimulus	Threshold Shift (dB) 14 Days after Noise Exposure						Threshold Shift (dB) 28 Days after Noise Exposure					
	Saline		RWS		USM		Saline		RWS		USM	
	Mean	SEM	Mean	SEM	Mean	SEM	Mean	SEM	Mean	SEM	Mean	SEM
8 kHz	56.7	2.11	50	4.28	43.3	4.22	53.3	2.11	43.3	2.47	32.5	2.81
16 kHz	44.2	3.00	36.7	3.33	27.5	2.50	43.3	3.07	33.3	2.11	24.2	1.54
24 kHz	38.3	2.11	26.7	1.67	24.2	3.00	35.8	1.54	26.7	2.11	23.3	2.11
32 kHz	35.8	3.27	31.7	3.58	29.2	5.07	35.8	2.39	25.8	2.39	24.2	4.17

## Data Availability

All relevant data are included within the manuscript. The raw data are available on request from the corresponding author.

## Sample Availability

Samples of the compounds are not available from the authors.
